# Iloprost infusion reduces serological cytokines and hormones of hypoxia and inflammation in systemic sclerosis patients

**DOI:** 10.1007/s10238-024-01374-4

**Published:** 2024-05-22

**Authors:** Chiara Pellicano, Amalia Colalillo, Oriana De Marco, Valeria Carnazzo, Umberto Basile, Antonietta Gigante, Rosario Cianci, Edoardo Rosato

**Affiliations:** 1 Department of Translational and Precision Medicine, Sapienza University of Rome, Viale Dell’Università 37, 00185 Rome, Italy; 2 Department of Public Health, Nephrology Unit, University Federico II, 80138 Naples, Italy; 3 UOC of Clinical Pathology DEA II Level, Hospital Santa Maria Goretti-ASL Latina, 04100 Latina, Italy

**Keywords:** Systemic sclerosis, NGAL, FGF-23, Klotho, Hypoxia

## Abstract

**Introduction:**

Systemic sclerosis (SSc) is characterized by microvascular damage of skin and internal organs with chronic hypoxia and release of cytokines and hormones such as neutrophil gelatinase-associated lipocalin (NGAL), fibroblast growth factor-23 (FGF-23) and Klotho. Aim of the study was to evaluate FGF-23, Klotho and NGAL serum levels in SSc patients and healthy controls (HC) and to evaluate serum levels changes of FGF-23, Klotho and NGAL after Iloprost.

**Methods:**

Twenty-one SSc patients and 20 HC were enrolled. In SSc patients, peripheral venous blood samples were collected at the first day before the autumn Iloprost infusion (t0), 60 min (t1) and 14 days after Iloprost infusion (t2).

**Results:**

SSc patients had higher serum level of FGF-23 [18.7 ± 6.4 pg/ml versus 3.6 ± 2.2 pg/ml, *p* < 0.001], Klotho [5.1 ± 0.8 pg/ml versus 2.3 ± 0.6 pg/ml, *p* < 0.001] and NGAL [20.9 ± 2.6 pg/ml versus 14.5 ± 1.7 pg/ml, *p* < 0.001] than HC.

Iloprost infusion reduces serum level of FGF-23 (18.7 ± 6.4 pg/ml versus 10.4 ± 5.5 pg/ml, *p* < 0.001), Klotho (5.1 ± 0.8 pg/ml versus 2.5 ± 0.6 pg/ml, *p* < 0.001) and NGAL (20.9 ± 2.6 pg/ml versus 15.1 ± 2.3 pg/ml, *p* < 0.001) between t0 and t1. The Iloprost infusion reduces serum level of FGF-23 (18.7 ± 6.4 pg/ml versus 6.6 ± 5.1 pg/ml), Klotho (5.1 ± 0.8 pg/ml versus 2.3 ± 0.4 pg/ml) and NGAL (20.9 ± 2.6 pg/ml versus 15.5 ± 1.9 pg/ml) between t0 and t2.

**Conclusions:**

SSc patients had higher FGF-23, Klotho and NGAL than HC. Iloprost reduces serum levels of FGF-23, Klotho and NGAL.

## Introduction

### Systemic sclerosis definition and pathogenesis

Systemic sclerosis (SSc) is an autoimmune disease characterized by dysregulation of immune system, endothelial dysfunction, microvascular damage and fibrosis of skin and internal organ [1].

The link between microvascular damage, endothelial dysfunction and development of dermal fibrosis is represented by the endothelial-to-mesenchymal transition (EndoMT) process: endothelial cells (ECs) dysfunction and their transition into myofibroblasts is responsible of early microvascular changes with cell injury and apoptosis that leads to immune system activation [1]. Microvascular damage is associated with chronic tissue hypoxia, oxidative stress, chronic inflammatory injury and release of angiogenic growth factors such as vascular endothelial growth factors (VEGF) [2–5]. In SSc, the angiogenesis is impaired and an imbalance of pro-angiogenic factors and angiogenesis inhibitors (endostatin and angiostatin) has been implicated in the progression of peripheral microvascular damage and fibrosis [4, 5]. The persistency of tissue damage and the increased and sustained release of cytokines and growth factors induces a progressive fibrotic state that involves tissues and organs [1].

### Microvascular complication in systemic sclerosis

In SSc, many complications such as digital ulcers (DUs), pulmonary arterial hypertension (PAH), scleroderma renal crisis (SRC) or renal scleroderma associated-vasculopathy, are due to microvascular damage. Raynaud’s phenomenon (RP) and DUs are the main vascular manifestation of skin involvement. Nailfold videocapillaroscopy (NVC) is the gold standard tool to evaluate microvascular damage of skin. Renal scleroderma associated-vasculopathy is characterized by vascular damage with normal renal function and increased intrarenal stiffness, assessed by renal resistive index (RRI) [6, 7]. Subclinical renal involvement is frequent in SSc patients and it is characterized by vascular damage in small and medium size vessels, probably due to renal Raynaud’s phenomenon vasospasm episodes, with consequent hypoxia, vasoconstriction and increase of RRI [6].

### Treatment of microvascular damage in systemic sclerosis

In SSc patients, antioxidant molecules (N-acetylcysteine) and Iloprost improve microvascular damage of skin and kidney [8–10]. Iloprost, a stable analogue of natural prostacyclin, is the gold standard treatment for skin vascular manifestation such as RP and DUs. Iloprost induces vasodilation, anti-platelet aggregation, cytoprotection, antioxidant effect and immunomodulation. These effects ameliorate the small vessel vasculopathy [11].

### Fibroblast growth factor-23, Klotho and neutrophil gelatinase-associated lipocalin

In hypoxic renal tissue, macrophagic infiltrates and damaged tubular cells release several inflammatory and profibrotic cytokines and chemokines such as neutrophil gelatinase-associated lipocalin (NGAL) [12]. NGAL is an acute-phase protein released by neutrophils and renal tubular cells in conditions of acute injury associated with inflammation and oxidative stress [13, 14]. NGAL have been proposed as signaling biomarkers of acute kidney injury (AKI) [15]. Changes in fibroblast growth factor-23 (FGF-23) and Klotho are also observed in course of hypoxia and inflammation [16, 17]. FGF-23 is a phosphotropic hormone secreted by osteoblasts and osteocytes that participates in the maintenance of mineral homeostasis; moreover, its role as an early biomarker for kidney dysfunction and as a predictor for risk of cardiovascular disease (CVD) has recently been established [18, 19]. Klotho acts either as an obligate coreceptor for FGF-23 or as a soluble pleiotropic endocrine hormone and it is mainly produced in the kidneys where is expressed in the distal and proximal convoluted tubules and the inner medullary collecting duct-derived cell lines [19, 20].

### Objectives of this study

Aim of this study was to evaluate at baseline serum levels of FGF-23, Klotho and NGAL in SSc patients and healthy controls (HC) and to correlate the serum levels of these markers with microvascular damage of skin and kidney. Secondary aim was to evaluate the serum levels of FGF-23, Klotho and NGAL after Iloprost infusion.

## Methods

### Subjects

Twenty-one consecutive SSc patients attending our Center, fulfilling the American College of Rheumatology/European League Against Rheumatism Collaborative Criteria for SSc [21], and 20 HC, matched for sex and age and recruited among healthcare workers, were enrolled in this study. All patients underwent treatment with calcium channel blockers and a single-day monthly Iloprost infusion for RP treatment.

In SSc patients, a two-month summer wash-out from Iloprost infusion before enrollment was an inclusion criteria. The wash-out period coincides with the routine interruption of the administration of Iloprost due to the summer period.

Exclusion criteria were: age < 18 years or > 70 years, cardiac failure, hepatic failure, end stage renal disease, PAH, systemic arterial hypertension, diabetes mellitus and other metabolic disorders, vitamin D deficiency, obstructive kidney disease or kidney diseases not related to SSc, immunosuppressive therapy and vitamin D supplementation in the last six months. We excluded SSc patients with cardiac failure, end renal stage disease or PAH from the study because it has been previously demonstrated that FGF-23 is expressed in cardiac myocytes and promotes cardiac metabolic remodeling in chronic kidney disease [22], moreover serum FGF-23 decreases in response to PAH-specific treatment [23]. Since NGAL have been proposed as signaling biomarkers of AKI we excluded patients with obstructive kidney disease or kidney diseases not related to SSc [15]. Finally, we excluded patients with metabolic disorders, vitamin D deficiency and vitamin D supplementation due the role of FGF-23 in bone metabolism [18, 19]. Smokers, pregnant and breastfeeding women were also excluded.

The subjects' written consent was obtained and the study was conducted according to the Declaration of Helsinki. The study was approved by the ethics committee of Sapienza University (IRB n 0486).

### Laboratory assays

Antibody profile was evaluated by indirect immunofluorescence assay (IFA) for the detection of ANA and definition of the immunofluorescence pattern (homogeneous, speckled, centromeric, cytoplasmic) [24], and by single enzyme-linked immunosorbent assay (ELISA) kits (MyBioSource, USA) for the detection of SSc-specific antibodies (Scl70, RNApolymeraseIII).

Peripheral venous blood samples were collected at the first day before the autumn Iloprost infusion (t0), 60 min after the end of the Iloprost infusion (t1) and 14 days after Iloprost infusion (t2). Peripheral venous blood was collected only at t0 for HC. Serum was separated from blood cells by centrifugation at 1500 × g for 15 min at room temperature and stored at − 80 °C until tests were performed. The three samples collected at the three different time points from each individual were concomitantly thawed and were determined at the same time using ELISA kits specific for Recombinant Human Klotho ELISA kits and Recombinant Human FGF-23 ELISA kits from (R&D Systems, McKinley Place, Minneapolis, MN, USA) and Human Lipocalin-2/NGAL Quantikine ELISA from (R&D Systems, McKinley Place, Minneapolis, MN, USA). Each sample was tested twice with an eight channel photometer-plate reader for sequential and simultaneous reading of micro-plate strips with 96 wells and 450 nm optical filter (DAS Srl, Italy) to minimize eventual discrepancies and all tests were performed in the same laboratory with the same instrument by the same expert operator without knowledge of the clinical informations of the handled samples. All safety precautions were taken while handling potentially harmful samples. Laboratory operators were all blind to the clinical information throughout the study.

### Clinical assessment of SSc patients

Clinical assessment of SSc patients has been performed by the same expert clinician with recording of subset of disease [diffuse cutaneous (dc)SSc or limited cutaneous (lc)SSc] and assessing modified Rodnan skin score (mRSS) [25]. Disease activity index (DAI) and disease severity scale (DSS) were also recorded [26, 27]. Nailfold videocapillaroscopy (NVC) has been performed with a videocapillaroscope (Pinnacle Studio Version 8) equipped with a 500 × optical probe. The patterns identified within the SSc pattern included early, active and late [28]. Capillaroscopic skin ulcers risk index (CSURI) is a validated tool to quantify microangiopathy. It was calculated as D x M/N^2^ (D-diameter of the biggest giant loop, M-number of giant loops, N-number of all loops), according to Sebastiani et al. [29]. Renal function was calculated using the Chronic Kidney Disease Epidemiology Collaboration (CKD-EPI) equation to estimate glomerular filtration rate (eGFR) [30]. Renal echocolordoppler ultrasound was performed to analyze blood velocity from the interlobar arteries by placing the probe at 3 different positions (mesorenal, superior, and inferior) and renal resistive index (RRI) was measured according to Rosato et al. [31]. According to 2022 ESC/ERS guidelines [32] we used the DETECT algorithm to select patients eligible for right heart catheterization (RHC) to confirm PAH diagnosis.

### Statistical analysis

SPSS version 26.0 software was used for statistical analysis. After evaluation of normality with Shapiro–Wilk’s test, continuous variables were expressed as mean and standard deviation (SD) or median and interquartile range (IQR), as appropriated. Student's *t*-test or Mann–Whitney's *U*-test were used to evaluate differences between groups. The Pearson or Spearman correlation test were used for bivariate correlations. A within-subjects one-way repeated-measures analysis of variance (ANOVA) was performed to compare the effect of Iloprost infusion on serum FGF-23, Klotho and NGAL in a cohort of 21 SSc patients. The sphericity was evaluated with Mauchly’s test and when the assumption of sphericity was violated, a Greenhouse-Geissen or Hyun-Feldt correction was used, as appropriated. A post hoc pairwise analysis using Bonferroni’s adjustement was performed for multiple comparisons and results were reported as mean and SD with mean difference and 95% confidence interval (95% CI). *p*-value < 0.05 was considered significant.

## Results

SSc patients have a median age of 56 years (IQR 49–60) with a median disease duration of 7 years (IQR 5–14). Table 1, shows the demographic and clinical characteristics of SSc patients.

**Table 1:** Demographic and clinical features of systemic sclerosis (SSc) patients. Continuous variables are expressed as median and interquartile range (IQR) and categorical variables are expressed as absolute frequency and percentage (%)

Age, years	56 (49–60)
Female/male	18 (85.7)/3 (14.3)
dcSSc/lcSSc	6 (28.6)/15 (71.4)
Disease duration	7 (5–14)
mRSS	10 (9–14)
Autoantibodies	
Anti-topoisomerase I	8 (38.1)
Anti-centromere	6 (28.6)
None	7 (33.3)
NVC	
Early	8 (38.1)
Active	7 (33.3)
Late	6 (28.6)
CSURI	1.89 (1.09–2.08)
≥ 2.94	3 (14.3)
DAI	1.2 (0.6–2.6)
DSS	7 (6–8.2)
DUs	6 (28.6)
PAH	0 (0)
ILD	13 (61.9)
GI	
GERD	21 (100)
SIBO	5 (23.8)
SRC	0 (0)
eGFR, ml/min	84 (76–95)
KDIGO classification	
I II IIIa	9 (42.8) 11 (52.4) 1 (4.8)
RRI	0.69 (0.64–0.71)
RRI ≥ 0.70	9 (42.8)

*SSc* Systemic Sclerosis, *dcSSc* Diffuse Cutaneous Systemic Sclerosis, *lcSSc* Limited Cutaneous Systemic Sclerosis, *mRSS* Modified Rodnan Skin Score, *NVC* Nailfold Videocapillaroscopy, *CSURI* Capillaroscopic Skin Ulcers Risk Index, *DAI* Disease Activity Index, *DSS* Disease Severity Scale, *DUs* digital ulcers, *PAH* Pulmonary Arterial Hypertension, *ILD* Interstitial Lung Disease, *GI* Gastrointestinal Involvement, *GERD* GastroEsophageal Reflux Disease, *SIBO* Small Intestinal Bacterial Overgrowth, *eGFR* Estimated Glomerular Filtration Rate, *RRI* Renal Resistive Index

### Serum levels of FGF-23, Klotho and NGAL at baseline

**Fig. 1:**
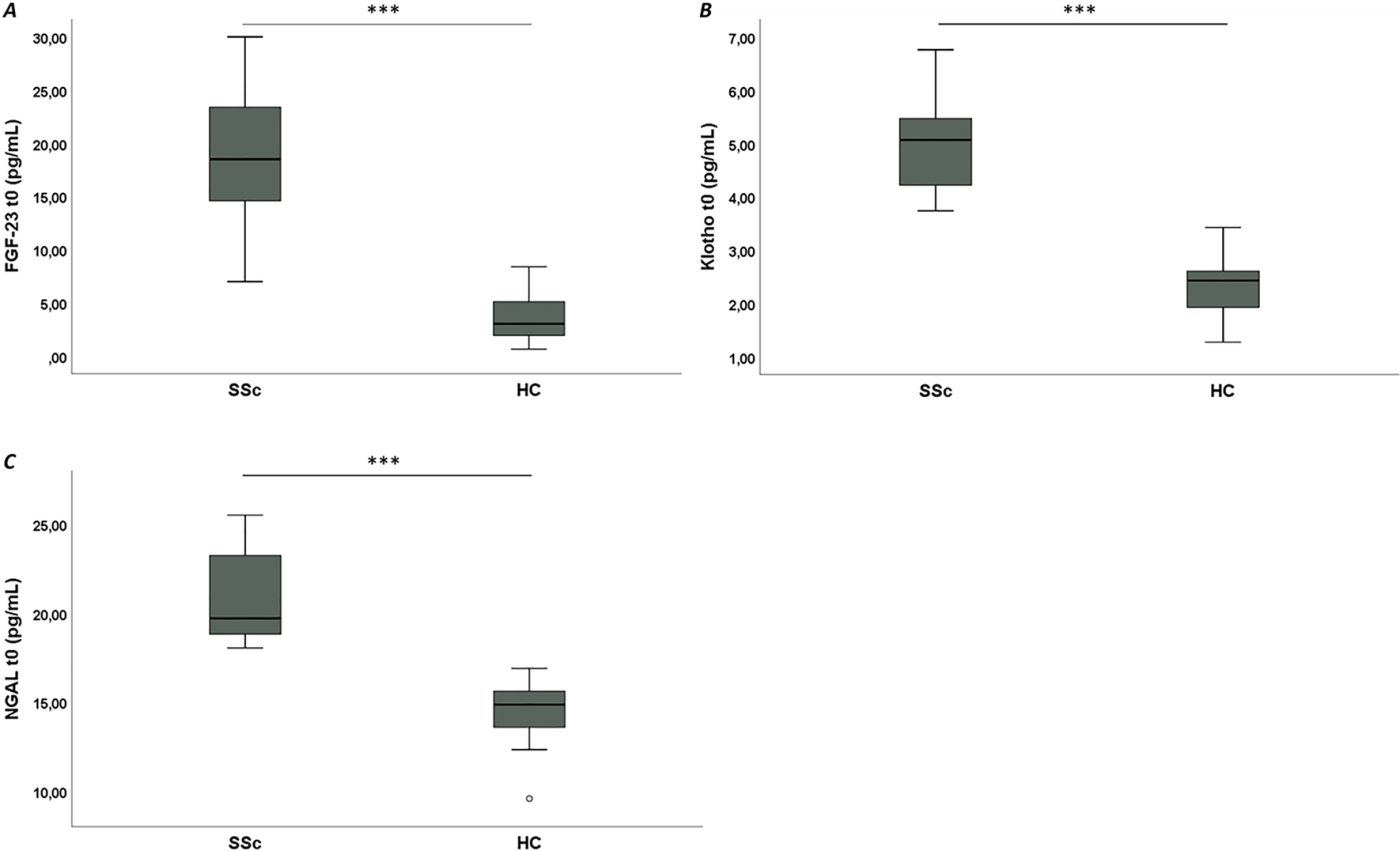
Comparative analysis at t0 between systemic sclerosis (SSc) patients and healthy controls (HC). **A**: Box plots show fibroblast growth factor-23 (FGF-23) serum levels; **B**: Box plots show Klotho serum levels; **C**: Box plots show neutrophil gelatinase-associated lipocalin (NGAL) serum levels. Circles are outliers. *p < 0.05, **p < 0.01, ***p < 0.001

At t0, SSc patients have significant higher serum levels of FGF-23 [18.7 ± 6.4 pg/ml versus 3.6 ± 2.2 pg/ml, *p* < 0.001, d = 3.1], Klotho [5.1 ± 0.8 pg/ml versus 2.3 ± 0.6 pg/ml, *p* < 0.001, d = 3.9] and NGAL [20.9 ± 2.6 pg/ml versus 14.5 ± 1.7 pg/ml, *p* < 0.001, d = 2.9] compared to HC (Fig. 1A-C).

NVC pattern is early in 8 (38.1%), active in 7 (33.3%) and late in 6 (28.6%) patients. FGF-23 serum levels does not show significant differences (*p* > 0.05) between SSc patients with early (21.17 ± 5.98 pg/ml), active (19.91 ± 5.07 pg/ml) and late (14 ± 6.86 pg/ml) NVC pattern. Klotho serum levels does not show significant differences (*p* > 0.05) in SSc patients with early (5.15 ± 1.05 pg/ml), active (5.09 ± 0.87 pg/ml) and late (4.97 ± 0.69 pg/ml) NVC pattern. NGAL serum levels does not show significant differences (*p* > 0.05) in SSc patients with early (21.28 ± 2.85 pg/ml), active (21.09 ± 2.94 pg/ml) and late (20.31 ± 2.41 pg/ml) NVC pattern.

We did not find any correlation between CSURI and serum levels of FGF-23 (r =  − 0.04, *p* > 0.05), Klotho (r = 0.2, *p* > 0.05) and NGAL (r = 0.158, *p* > 0.05); moreover, serum levels of FGF-23 (14.19 ± 6.86 pg/ml versus 19.73 ± 6.59 pg/ml), Klotho (5.71 ± 0.55 pg/ml versus 4.98 ± 0.91 pg/ml) and NGAL (20.48 ± 1.08 pg/ml versus 20.48 ± 2.54 pg/ml) were similar (*p* > 0.05) between patients with CSURI ≥ 2.94 and patients with CSURI < 2.94.

**Fig. 2:**
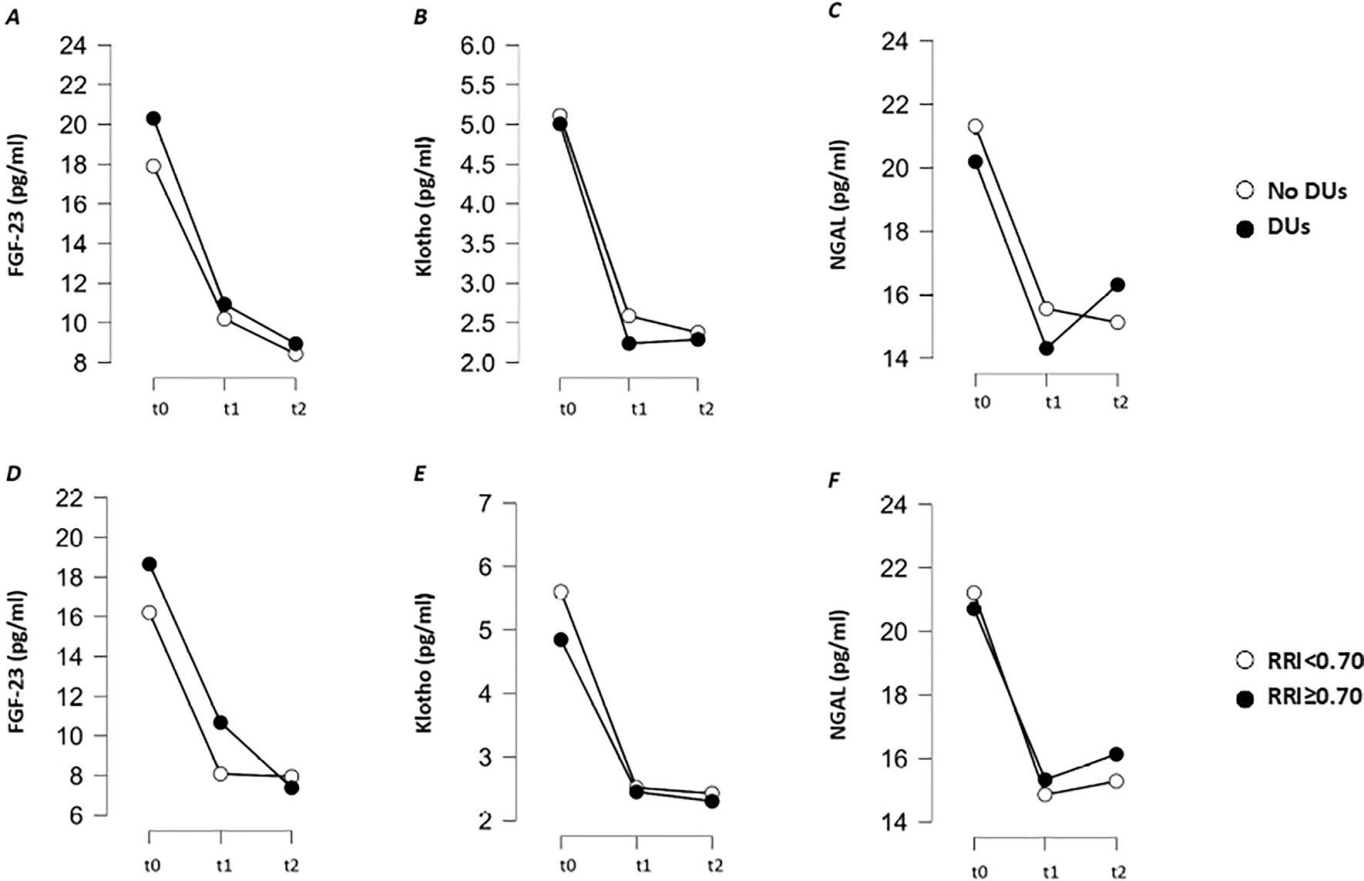
Changes at three different time points (t0, t1, t2) in systemic sclerosis (SSc) patients. **A**: Comparative analysis of fibroblast growth factor-23 (FGF-23) serum levels at each time point between SSc patients with or without digital ulcers (DUs); **B**: Comparative analysis of Klotho serum levels at each time point between SSc patients with or without DUs; **C**: Comparative analysis of neutrophil gelatinase-associated lipocalin (NGAL) serum levels at each time point between SSc patients with or without DUs; **D**: Comparative analysis of FGF-23 serum levels at each time point between SSc patients with normal or increased renal resistive index (RRI); E: Comparative analysis of Klotho serum levels at each time point between SSc patients with normal or increased RRI; F: Comparative analysis of NGAL serum levels at each time point between SSc patients with normal or increased RRI

Six (28.6%) patients have DUs. There were no statistically significant differences (*p* > 0.05) in serum level of FGF-23 [20.31 ± 5.95 pg/ml versus 17.9 ± 6.75 pg/ml], Klotho [5.01 ± 0.97 pg/ml versus 5.11 ± 0.83 pg/ml] and NGAL [20.19 ± 2.35 pg/ml versus 21.31 ± 2.8 pg/ml] between SSc patients with DUs and SSc patients without DUs (Fig. 2A-C).

The median value of eGFR is 84 ml/min (IQR 76–95). At t0, serum levels of FGF-23 (r =  − 0.325, *p* > 0.05), Klotho (r = 0.387, *p* > 0.05) and NGAL (r = 0.136, *p* > 0.05) do not show a significant correlation with eGFR.

Median RRI is 0.69 (IQR 0.64–0.71) and 9 (42.8%) SSc patients have RRI ≥ 0.70. At t0, serum levels of FGF-23 (r = 0.278, *p* > 0.05), Klotho (r =  − 0.241, *p* > 0.05) and NGAL (r =  − 0.166, *p* > 0.05) do not show a significant correlation with RRI. At t0, there are no significant differences of FGF-23 [18.6 ± 4.7 pg/ml versus 16.2 ± 6.9 pg/ml, *p* > 0.05], Klotho [4.8 ± 0.9 pg/ml versus 5.6 ± 0.6 pg/ml, *p* > 0.05] and NGAL [20.7 ± 2.9 pg/ml versus 21.2 ± 2.4 pg/ml, *p* > 0.05] in SSc patients with RRI ≥ 0.70 compared to SSc patients with normal RRI (Fig. 2D-F).

### Serum levels of FGF-23, Klotho and NGAL after Iloprost infusion

**Fig. 3:**
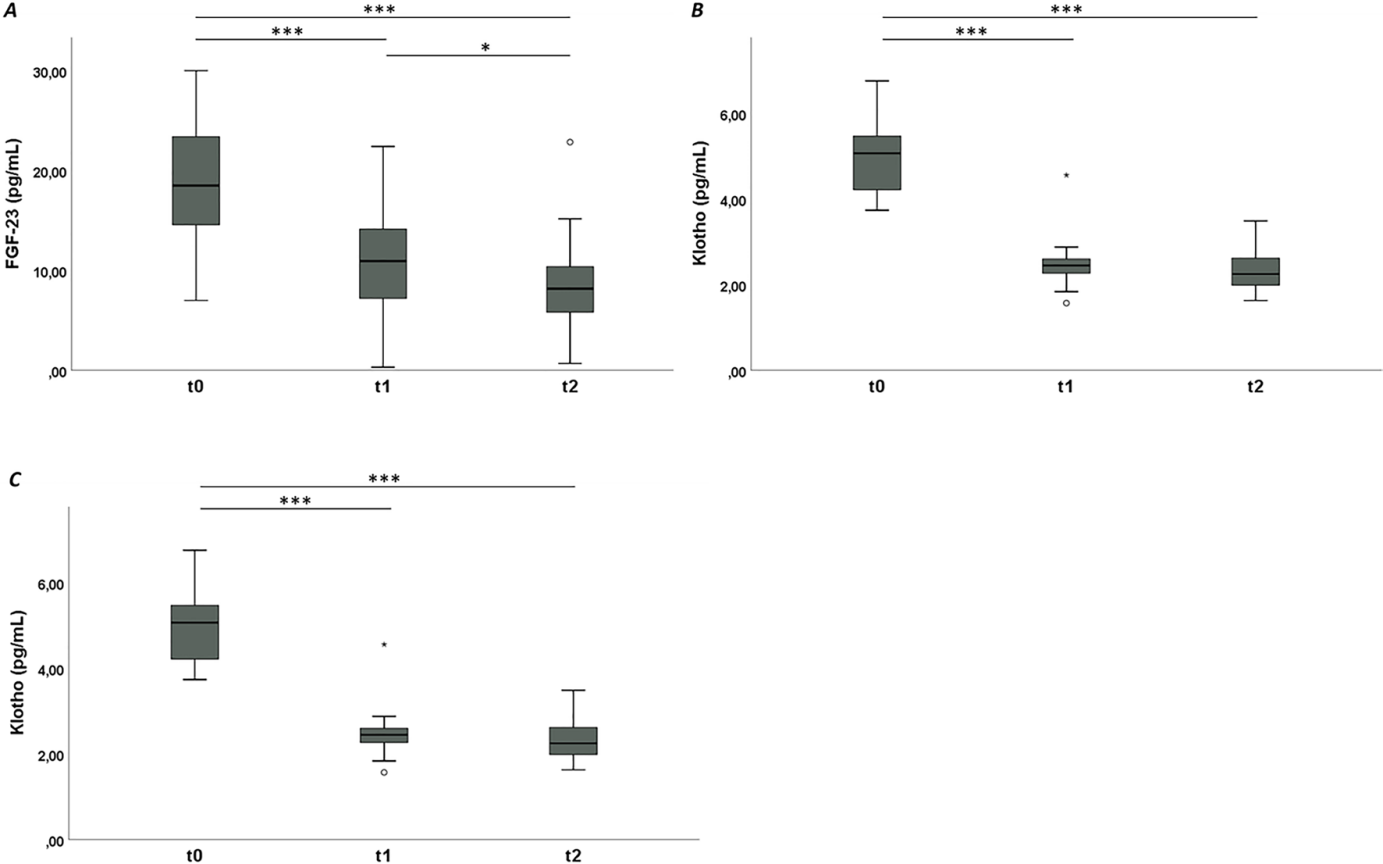
Repeated measures at three different time points (t0, t1, t2) in systemic sclerosis (SSc) patients. **A**: Box plots show fibroblast growth factor-23 (FGF-23) serum levels; **B**: Box plots show Klotho serum levels; **C**: Box plots show neutrophil gelatinase-associated lipocalin (NGAL) serum levels. Circles are outliers. *p < 0.05, **p < 0.01, ***p < 0.001

The mean value of FGF-23 is significantly higher at t0 (18.7 ± 6.4 pg/ml) compared to t1 (10.4 ± 5.5 pg/ml) and t2 (8.6 ± 5.1 pg/ml) and also at t1 compared to t2 (Table 2 and Fig. 3A).

**Table 2:** Means (M) and standard deviations (SD) for fibroblast growth factor-23 (FGF-23), Klotho and neutrophil gelatinase-associated lipocalin (NGAL) at each time point and post hoc pairwise analysis using Bonferroni’s adjustment for multiple comparisons

Descriptives		Post hoc test		
Time point	M ± SD	Comparison	Mean difference (95% CI)	*p*
FGF-23 (pg/ml)				
t0	18.7 ± 6.4	t0-t1	8.255 (6.004;10.505)	< 0.001
t1	10.4 ± 5.5	t0-t2	10.092 (7.842;12.342)	< 0.001
t2	8.6 ± 5.1	t1-t2	1.837 (0.413;4.008)	< 0.05
Klotho (pg/ml)				
t0	5.1 ± 0.8	t0-t1	2.605 (2.215;2.995)	< 0.001
t1	2.5 ± 0.6	t0-t2	2.728 (2.338;3.118)	< 0.001
t2	2.3 ± 0.4	t1-t2	0.123 (− 0.267;0.513)	> 0.05
NGAL (pg/ml)				
t0	20.9 ± 2.6	t0-t1	5.799 (4.144;7.453)	< 0.001
t1	15.1 ± 2.3	t0-t2	5.420 (3.766;7.074)	< 0.001
t2	15.5 ± 1.9	t1-t2	− 0.379 (− 2.033;1.279)	> 0.05

The mean value of Klotho is significantly higher at t0 (5.1 ± 0.8 pg/ml) compared to t1 (2.5 ± 0.6 pg/ml) and t2 (2.3 ± 0.4 pg/ml) (Table 2 and Fig. 3B). There is no statistically significant difference in Klotho serum level between t1 and t2 (Table 2).

The mean value of NGAL is significantly higher at t0 (20.9 ± 2.6 pg/ml) compared to t1 (15.1 ± 2.3 pg/ml) and t2 (15.5 ± 1.9 pg/ml) (Table 2 and Fig. 3C). There is no statistically significant difference in NGAL serum level between t1 and t2 (Table 2).

SSc patients have significant higher serum levels of FGF-23 after Iloprost infusion, both at t1 and t2, compared to HC [10.4 ± 5.5 pg/ml and 8.6 ± 5.1 pg/ml versus 3.6 ± 2.2 pg/ml, *p* < 0.001]. We did not find significant difference (*p* > 0.05) in Klotho serum level between SSc patients after Iloprost infusion, both at t1 (2.5 ± 0.6 pg/ml) and t2 (2.3 ± 0.4 pg/ml), and HC (2.3 ± 0.6 pg/ml). We did not find significant difference (*p* > 0.05) in NGAL serum level between SSc patients after Iloprost infusion, both at t1 (15.1 ± 2.3 pg/ml) and t2 (15.5 ± 1.9 pg/ml), and HC (14.5 ± 1.7 pg/ml).

## Discussion

In this study, we found that SSc patients had higher serum level of FGF-23, Klotho and NGAL than HC and Iloprost infusion reduces significantly their serum level.

In SSc patients, microvascular damage with chronic hypoperfusion is the main pathogenic mechanism of skin and internal organs damage. Many complications of SSc (RP, DUs, PAH, SRC) are due to vascular involvement and vasoactive therapies were used to slow the progression of vasculophathy. SSc patients have a chronic hypoperfusion that improves after Iloprost infusion, both in the skin and in the internal organs [8, 9]. Furthermore, Iloprost has been shown to have a long-lasting endothelium protective effect [11]. Prolonged hypoperfusion contributes to activation of inflammatory signals and oxidative stress, thereby accelerating injury and fibrosis in skin and kidney. It has been demonstrated a change in inflammatory biomarkers, including NGAL, after renal revascularization in patients with atherosclerotic renal artery stenosis (ARAS) [33]. In patients with ARAS, peripheral levels of NGAL fell after restoration of vessel patency and remained below baseline for 24 h; over the subsequent 3 months, this marker returned to baseline levels [33]. The Authors interpret these findings to indicate that processes of ongoing tissue injury and inflammation persist.

In this study, we demonstrated that FGF-23, Klotho and NGAL were increased in SSc patients compared to HC. We can suppose that persistent state of hypoxia leads to inflammation and microvascular damage of skin and internal organs.

In literature, there are few data about the differences in serum concentrations of FGF-23 and Klotho in SSc patients compared to HC [34–40]. Cantero-Nieto et al. [34] showed similar serum FGF-23 between SSc patients and HC with higher FGF-23 in SSc patients with calcinosis compared to SSc patients without calcinosis. Ahmadi et al. [35] found similar FGF-23 and lower Klotho in SSc patients compared to HC, whilst Kotyla et al. [36] found reduced levels of FGF-23 and similar levels of Klotho in SSc patients compared to HC. In addition, these authors demonstrated that FGF-23/Klotho index correlated with DAI. In contrast with these data, we found higher FGF-23 and Klotho serum level in SSc patients compared to HC. This could be due to different patient selection: we excluded SSc patient with vitamin D deficency which is known to contribute to a reduction of Klotho; moreover, the difference of our results compared to data literature may be due to the interruption of vasoactive treatment before the laboratory assessment. to minimize the possible interference of Iloprost, as Klotho seems to play an essential role in the maintenance of vessel tone control. Previous studies demonstrated that Klotho contrasts FGF-23 mediated vasoconstriction, by increasing the production of nitric oxide (NO) and indirectly regulating the intracellular calcium concentration by binding to the complex VEGFR/TRPC-1 on ECs [41, 42]. We may hypothesize that the higher Klotho levels found in SSc patients are due to the compensatory mechanism to maintain vessel tone and to contrast FGF-23 mediated vasoconstriction.

In literature, there are few data about the differences in serum concentrations of NGAL in SSc patients compared to HC. Takahashi et al. [43] found comparable NGAL serum levels between SSc patients and HC. The prevalence of SRC was significantly higher in SSc patients with elevated serum NGAL than in those with normal levels and NGAL serum levels inversely correlated with eGFR in SSc patients with renal dysfunction [43]. Microvascular damage is a hallmark of SSc and it plays a key role in the pathogenesis of internal organs damage [1]. Renal scleroderma associated vasculopathy is characterized by increased RRI that over the time leads to parenchymal thickness reduction with atrophy and worsening of renal function, assessed by eGFR [44]. Renal ischemia–reperfusion injury is the primary cause of AKI, which can cause a significant increase in the expression of NGAL in kidneys with consequent NGAL accumulation in the blood and urine, which can be detected in patients with AKI [45]. In AKI, NGAL is not only a serum/urinary marker of its severity but also a central effector of progressive renal tissue damage [45]. In renovascular disease, NGAL is predictor of outcome after revascularization especially in patients with increased RRI [46]. In our study, we did not find any difference in FGF-23, Klotho and NGAL serum levels between SSc patients with increased RRI compared to SSc patients with normal RRI. In addition, we did not find any correlation between FGF-23, Klotho and NGAL serum level and eGFR. We can hypothesize that FGF-23, Klotho and NGAL are markers of chronic hypoxia and inflammation due to microvascular damage of skin and internal organs. Elevated level of NGAL are present in patients with acute vascular renal injury (renal arterial stenosis and SRC). SRC is an acute vascular complication of SSc. In these conditions, NGAL is also a predictive marker of outcome.

For the first time, we demonstrated that Iloprost infusion reduces significantly serum level of FGF-23, Klotho and NGAL. In literature, there are no data about serum changes of these markers before and after vasoactive treatment. Iloprost has a key role in skin (RP and DUs) and renal vasculopathy in SSc patients. Scorza et al. [8] demonstrated that Iloprost infusion reduces RRI and improves renal perfusion in SSc patients. We can hypothesize that chronic hypoxic state leads to release hypoxia-related inflammation biomarkers such as FGF-23, Klotho and NGAL. It is well-known that hypoxia is a landmark common to all CVD (hypertension, arterial aneurysms, atherosclerosis, PAH, congestive heart failure, etc.), and hypoxia-inducible factors (HIF) are involved in many mechanisms such as erythropoiesis, angiogenesis, and inflammation. Any therapeutic approach addressed at stabilizing HIF activity might have a potential benefit in diseases with microvascular injury such as SSc vasculopathy.

Finally, we demonstrated that Iloprost treatment “normalizes” the serum levels of Klotho and NGAL but not of FGF-23 which is however reduced. As previously showed, FGF-23 is a biologically active hormone that plays a key role in the complex network between the bones and other organs and it could be involved in the mechanism of soft tissue calcification [34]. Further evidence links FGF-23 to vasoconstriction, decreased NO availability and oxidative stress, which lead to vascular smooth muscle cell and endothelial dysfunction [47]. We may hypothesize that in SSc patients FGF-23 production is increased due to chronic tissue hypoxia and oxidative stress, secondary to microvascular damage. Although Iloprost infusion reduces serum level of FGF-23, it is not sufficient to restore a normal level of this hormone. Therefore, FGF-23 could be a possible therapeutic target for future treatments.

Main limitations of this study included the monocentric design, small sample size, lack of assessment of urinary FGF-23, Klotho and NGAL. Further research is needed to demonstrate the role of NGAL in association with other innovative biomarkers of ischemia–reperfusion injury such as FGF-23 and Klotho in SSc patients.

In conclusion, FGF-23, Klotho and NGAL are increased in SSc patients for chronic hypoxia due to microvascular damage. Iloprost ameliorates tissue hypoperfusion and hypoxia with reduction of serum level of FGF-23, Klotho and NGAL. Future large studies are need to evaluate these preliminary data.

## Data Availability

All data are present in the manuscript.

## References

[1] Cutolo M, Soldano S, Smith V. Pathophysiology of systemic sclerosis: current understanding and new insights. Expert Rev Clin Immunol. 2019;15:753–64. 10.1080/1744666X.2019.1614915. 31046487 10.1080/1744666X.2019.1614915

[2] Distler JH, Gay S, Distler O, Angiogenesis and vasculogenesis in systemic sclerosis, Rheumatology (Oxford), 2006; 45 (Suppl 3), pp iii26–27. doi: 10.1093/rheumatology/kel295. Erratum in: Rheumatology (Oxford), 2008; 47, 234–235. 10.1093/rheumatology/kel295 16987827

[3] Flower VA, Barratt SL, Ward S, Pauling JD. The Role of vascular endothelial growth factor in systemic sclerosis. Curr Rheumatol Rev. 2019;15:99–109. 10.2174/1573397114666180809121005. 30091416 10.2174/1573397114666180809121005

[4] Liakouli V, Cipriani P, Marrelli A, Alvaro S, Ruscitti P, Giacomelli R. Angiogenic cytokines and growth factors in systemic sclerosis. Autoimmun Rev. 2011;10:590–4. 10.1016/j.autrev.2011.04.019. 21549861 10.1016/j.autrev.2011.04.019

[5] Almeida I, Oliveira Gomes A, Lima M, Silva I, Vasconcelos C. Different contributions of angiostatin and endostatin in angiogenesis impairment in systemic sclerosis: a cohort study. Clin Exp Rheumatol. 2016;34(Suppl 100):37–42. 26885625

[6] Gigante A, Barbano B, Gasperini ML, Zingaretti V, Cianci R, Rosato E. Renal parenchymal thickness in patients with systemic sclerosis is related to intrarenal hemodynamic variables and raynaud renal phenomenon. J Rheumatol. 2020;47:567–71. 10.3899/jrheum.190165. 31203218 10.3899/jrheum.190165

[7] Shanmugam VK, Steen VD. Renal manifestations in scleroderma: evidence for subclinical renal disease as a marker of vasculopathy. Int J Rheumatol. 2010;2010:538589. 10.1155/2010/538589. 20827302 10.1155/2010/538589 PMC2933853

[8] Scorza R, Rivolta R, Mascagni B, Berruti V, Bazzi S, Castagnone D, Quarto di Palo F. Effect of iloprost infusion on the resistance index of renal vessels of patients with systemic sclerosis. J Rheumatol. 1997;24:1944–8. 9330936

[9] Scorza R, Caronni M, Mascagni B, Berruti V, Bazzi S, Micallef E, Arpaia G, Sardina M, Origgi L, Vanoli M. Effects of long-term cyclic iloprost therapy in systemic sclerosis with Raynaud’s phenomenon. A randomized, controlled study. Clin Exp Rheumatol. 2001;19:503–8. 11579708

[10] Rosato E, Cianci R, Barbano B, Menghi G, Gigante A, Rossi C, Zardi EM, Amoroso A, Pisarri S, Salsano F. N-acetylcysteine infusion reduces the resistance index of renal artery in the early stage of systemic sclerosis. Acta Pharmacol Sin. 2009;30:1283–8. 10.1038/aps.2009.128. 19730428 10.1038/aps.2009.128 PMC4007187

[11] Giordo R, Thuan DTB, Posadino AM, Cossu A, Zinellu A, Erre GL, Pintus G. Iloprost attenuates oxidative stress-dependent activation of collagen synthesis induced by sera from scleroderma patients in human pulmonary microvascular endothelial cells. Molecules. 2001;26:4729. 10.3390/molecules26164729. 10.3390/molecules26164729 PMC8399120 34443317

[12] Cianci R, Simeoni M, Cianci E, De Marco O, Pisani A, Ferri C, Gigante A. Stem cells in kidney ischemia: from inflammation and fibrosis to renal tissue regeneration. Int J Mol Sci. 2023;24:4631. 10.3390/ijms24054631. 36902062 10.3390/ijms24054631 PMC10002584

[13] Mellor A, Boos C, Stacey M, Hooper T, Smith C, Begley J, Yarker J, Piper R, O’Hara J, King R, Turner S, Woods DR. Neutrophil gelatinase-associated lipocalin: its response to hypoxia and association with acute mountain sickness. Dis Markers. 2013;35:537–42. 10.1155/2013/601214. 24227892 10.1155/2013/601214 PMC3817649

[14] Bolignano D, Donato V, Coppolino G, Campo S, Buemi A, Lacquaniti A, Buemi M. Neutrophil gelatinase-associated lipocalin (NGAL) as a marker of kidney damage. Am J Kidney Dis. 2008;52:595–605. 10.1053/j.ajkd.2008.01.020. 18725016 10.1053/j.ajkd.2008.01.020

[15] Wang W, Saad A, Herrmann SM, Eirin Massat A, McKusick MA, Misra S, Lerman LO, Textor SC. Changes in inflammatory biomarkers after renal revascularization in atherosclerotic renal artery stenosis. Nephrol Dial Transplant. 2016;31:1437–43. 10.1093/ndt/gfv448. (**Epub 2016 Jan 29**). 26908767 10.1093/ndt/gfv448 PMC5009289

[16] Afsar B, Kanbay M, Afsar RE. Interconnections of fibroblast growth factor 23 and klotho with erythropoietin and hypoxia-inducible factor. Mol Cell Biochem. 2022;477:1973–85. 10.1007/s11010-022-04422-3. 35381946 10.1007/s11010-022-04422-3

[17] Francis C, David V. Inflammation regulates fibroblast growth factor 23 production. Curr Opin Nephrol Hypertens. 2016;25:325–32. 10.1097/MNH.0000000000000232. 27191351 10.1097/MNH.0000000000000232 PMC5016608

[18] Ho BB, Bergwitz C. FGF23 signalling and physiology. J Mol Endocrinol. 2021;66:R23–32. 10.1530/JME-20-0178. 33338030 10.1530/JME-20-0178 PMC8782161

[19] Lu X, Hu MC. Klotho/FGF23 axis in chronic kidney disease and cardiovascular disease. Kidney Dis (Basel). 2017;3:15–23. 10.1159/000452880. 28785560 10.1159/000452880 PMC5527179

[20] Muñoz-Castañeda JR, Rodelo-Haad C, Pendon-Ruiz de Mier MV, Martin-Malo A, Santamaria R, Rodriguez M. Klotho/FGF23 and Wnt signaling as important players in the comorbidities associated with chronic kidney disease. Toxins (Basel). 2020;12:185. 10.3390/toxins12030185. 32188018 10.3390/toxins12030185 PMC7150840

[21] van den Hoogen F, Khanna D, Fransen J, Johnson SR, Baron M, Tyndall A, Matucci-Cerinic M, Naden RP, Jr Medsger TA, Carreira PE, Riemekasten G, Clements PJ, Denton CP, Distler O, Allanore Y, Furst DE, Gabrielli A, Mayes MD, van Laar JM, Seibold JR, Czirjak L, Steen VD, Inanc M, Kowal-Bielecka O, Müller-Ladner U, Valentini G, Veale DJ, Vonk MC, Walker UA, Chung L, Collier DH, Ellen Csuka M, Fessler BJ, Guiducci S, Herrick A, Hsu VM, Jimenez S, Kahaleh B, Merkel PA, Sierakowski S, Silver RM, Simms RW, Varga J, Pope JE. classification criteria for systemic sclerosis: an American college of rheumatology/European league against rheumatism collaborative initiative. Ann Rheum Dis. 2013;72(2013):1747–55. 10.1136/annrheumdis-2013-204424. 24092682 10.1136/annrheumdis-2013-204424

[22] Figurek A, Rroji M, Spasovski G. FGF23 in chronic kidney disease: bridging the heart and anemia. Cells. 2023;12:609. 10.3390/cells1204609. 36831276 10.3390/cells12040609 PMC9954184

[23] Bouzina H, Hesselstrand R, Rådegran G. Higher plasma fibroblast growth factor 23 levels are associated with a higher risk profile in pulmonary arterial hypertension. Pulm Circ. 2019;27:2045894019895446. 10.1177/2045894019895446. 10.1177/2045894019895446 PMC6935881 31908768

[24] Chan EK, Damoiseaux J, de Melo Cruvinel W, Carballo OG, Condrad K, Francescantonio PL, Fritzler MJ, Garcia-De La Torre I, Herold M, Mimori T, Satoh M, von Mühlen CA, Andrade LE. Report on the second international consensus on ANA pattern (ICAP) workshop in Dresden 2015. Lupus. 2016;25:797–804. 10.1177/0961203316640920. 27252255 10.1177/0961203316640920

[25] LeRoy EC, Black C, Fleischmajer R, Jablonska S, Krieg T, MedsgerJr TA, Rowell N, Wollheim F. Scleroderma (systemic sclerosis): classification, subsets and pathogenesis. J Rheumatol. 1988;15:202–5. 3361530

[26] Valentini G, Iudici M, Walker UA, Jaeger VK, Baron M, Carreira P, Czirják L, Denton CP, Distler O, Hachulla E, Herrick AL, Kowal-Bielecka O, Pope J, Müller-Ladner U, Riemekasten G, Avouac J, Frerix M, Jordan S, Minier T, Siegert E, Ong VH, Vettori S, Allanore Y. The European scleroderma trials and research group (EUSTAR) task force for the development of revised activity criteria for systemic sclerosis: derivation and validation of a preliminarily revised EUSTAR activity index. Ann Rheum Dis. 2017;76:270–6. 10.1136/annrheumdis-2016-209768. 27621285 10.1136/annrheumdis-2016-209768

[27] Jr Medsger TA, Silman AJ, Steen VD, Black CM, Akesson A, Bacon PA, Harris CA, Jablonska S, Jayson MI, Jimenez SA, Krieg T, Leroy EC, Maddison PJ, Russell ML, Schachter RK, Wollheim FA, Zacharaie H. A disease severity scale for systemic sclerosis: development and testing. J Rheumatol. 1999;26:2159–67. 10529133

[28] Cutolo M, Matucci Cerinic M. Nailfold capillaroscopy and classification criteria for systemic sclerosis. Clin Exp Rheumatol. 2007;25:663–5. 18078610

[29] Sebastiani M, Manfredi A, Vukatana G, Moscatelli S, Riato L, Bocci M, Iudici M, Principato A, Mazzuca S, Del Medico P, De Angelis R, D’Amico R, Vicini R, Colaci M, Ferri C. Predictive role of capillaroscopic skin ulcer risk index in systemic sclerosis: a multicentre validation study. Ann Rheum Dis. 2012;71:67–70. 10.1136/annrheumdis-2011-200022. 21917823 10.1136/annrheumdis-2011-200022

[30] Gigante A, Rosato E, Massa R, Rossi C, Barbano B, Cianci R, Molinaro I, Amoroso A, Salsano F. Evaluation of chronic kidney disease epidemiology collaboration equation to estimate glomerular filtration rate in scleroderma patients. Rheumatology (Oxford). 2012;51:1426–31. 10.1093/rheumatology/kes049. 22457437 10.1093/rheumatology/kes049

[31] Rosato E, Gigante A, Barbano B, Cianci R, Molinaro I, Rossi C, Massa R, Amoroso A, Pisarri S, Salsano F. Intrarenal hemodynamic parameters correlate with glomerular filtration rate and digital microvascular damage in patients with systemic sclerosis. Semin Arthritis Rheum. 2012;41:815–21. 10.1016/j.semarthrit.2011.11.005. 22192932 10.1016/j.semarthrit.2011.11.005

[32] Humbert M, Kovacs G, Hoeper MM, Badagliacca R, Berger RMF, Brida M, Carlsen J, Coats AJS, Escribano-Subias P, Ferrari P, Ferreira DS, Ghofrani HA, Giannakoulas G, Kiely DG, Mayer E, Meszaros G, Nagavci B, Olsson KM, Pepke-Zaba J, Quint JK, Rådegran G, Simonneau G, Sitbon O, Tonia T, Toshner M, Vachiery JL, Vonk Noordegraaf A, Delcroix M, Rosenkranz S; ESC/ERS Scientific Document Group. 2022 ESC/ERS Guidelines for the diagnosis and treatment of pulmonary hypertension. Eur Heart J, 2022; 43, 3618–3731. doi: 10.1093/eurheartj/ehac237. Erratum in: Eur Heart J, 2023; 44, 1312

[33] Wang W, Saad A, Herrmann SM, Eirin Massat A, McKusick MA, Misra S, Lerman LO, Textor CS. Changes in inflammatory biomarkers after renal revascularization in atherosclerotic renal artery stenosis. Nephrol Dial Transplant. 2016;31:1437–43. 10.1093/ndt/gfv448. 26908767 10.1093/ndt/gfv448 PMC5009289

[34] Cantero-Nieto L, Alvarez-Cienfuegos A, García-Gómez JA, Martin J, González-Gay MA, Ortego-Centeno N. Role of fibroblast growth factor-23 in calcinosis in women with systemic sclerosis. Acta Reumatol Port. 2020;45:259–64. 33420766

[35] Ahmadi R, Hajialilo M, Ghorbanihaghjo A, Mota A, Raeisi S, Bargahi N, Valilo M, Askarian F. FGF-23, Klotho and vitamin D levels in scleroderma. Iran J Public Health. 2017;46:530–6. 28540270 PMC5439043

[36] Kotyla PJ, Kruszec-Zytniewska A, Owczarek AJ, Olszanecka-Glinianowicz M, Chudek J. Fibroblast growth factor 23 to Alpha-Klotho index correlates with systemic sclerosis activity: a proposal for novel disease activity marker. J Clin Med. 2018;7:558. 10.3390/jcm7120558. 30562918 10.3390/jcm7120558 PMC6306722

[37] Talotta R, Bongiovanni S, Letizia T, Rigamonti F, Atzeni F, Benucci M, Vago T, Sarzi-Puttini P. The role of klotho in systemic sclerosis. Reumatismo. 2017;69:156–63. 10.4081/reumatismo.2017.987. 29320841 10.4081/reumatismo.2017.987

[38] Talotta R, Bongiovanni S, Letizia T, Rigamonti F, Ditto MC, Atzeni F, Salaffi F, Batticciotto A, Gerardi MC, Antivalle M, Vago T, Benucci M, Sarzi-Puttini P. Measurement of serum Klotho in systemic sclerosis. Dis Markers. 2017;2017:9545930. 10.1155/2017/9545930. 28912623 10.1155/2017/9545930 PMC5585626

[39] Niazy MH, Gaber W, Sayed S, Shaker OG, Gheita TA. The anti-aging protein alpha-Klotho in systemic sclerosis patients: does a relationship to telangiectasia exist? Z Rheumatol. 2020;79:404–9. 31602506 10.1007/s00393-019-00718-w

[40] Hajialilo M, Noorabadi P, Tahsini Tekantapeh S, Malek Mahdavi A. Endothelin-1, α-Klotho, 25(OH) Vit D levels and severity of disease in scleroderma patients. Rheumatol Int. 2017;37:1651–7. 10.1007/s00296-017-3797-z. 28831601 10.1007/s00296-017-3797-z

[41] Six I, Okazaki H, Gross P, et al. Direct, acute effects of Klotho and FGF23 on vascular smooth muscle and endothelium. PLoS One. 2014;9:e93423. 24695641 10.1371/journal.pone.0093423 PMC3973676

[42] Kusaba T, Okigadi M, Matui A, et al. Klotho is associated with VEGF receptor-2 and the transient receptor potential canonical-1 Ca2+ channel to maintain endothelial integrity. Proc Natl Acad Sci USA. 2010;107:19308–13. 10.1073/pnas.1008544107. 20966350 10.1073/pnas.1008544107 PMC2984167

[43] Takahashi T, Asano Y, Noda S, Aozasa N, Akamata K, Taniguchi T, Ichimura Y, Toyama T, Sumida H, Kuwano Y, Tada Y, Sugaya M, Kadono T, Sato S. A possible contribution of lipocalin-2 to the development of dermal fibrosis, pulmonary vascular involvement and renal dysfunction in systemic sclerosis. Br J Dermatol. 2015;173:681–9. 10.1111/bjd.13779. 25781362 10.1111/bjd.13779

[44] Gigante A, Leodori G, Pellicano C, Villa A, Rosato E. Assessment of kidney involvement in systemic sclerosis: from scleroderma renal crisis to subclinical renal vasculopathy. Am J Med Sci. 2002;364:529–37. 10.1016/j.amjms.2022.02.014. 10.1016/j.amjms.2022.02.014 35537505

[45] Haase M, Bellomo R, Devarajan P, Schlattmann P, Haase-Fielitz A. NGAL Meta-analysis investigator group, accuracy of neutrophil gelatinase-associated lipocalin (NGAL) in diagnosis and prognosis in acute kidney injury: a systematic review and meta-analysis. Am J Kidney Dis. 2009;54:1012–24. 19850388 10.1053/j.ajkd.2009.07.020

[46] Cianci R, Simeoni M, Gigante A, Marco Perrotta A, Ronchey S, Mangialardi N, Schioppa A, De Marco O, Cianci E, Barbati C, Lai S, Ferri C. Renal stem cells, renal resistive index, and neutrophil gelatinase associated lipocalin changes after revascularization in patients with renovascular hypertension and ischemic nephropathy. Curr Pharm Des. 2023;29:133–8. 10.2174/1381612829666221213104945. 36515041 10.2174/1381612829666221213104945

[47] Silswal N, Touchberry CD, Daniel DR, McCarthy DL, Zhang S, Andresen J, Stubbs JR, Wacker MJ. FGF23 directly impairs endothelium-dependent vasorelaxation by increasing superoxide levels and reducing nitric oxide bioavailability. Am J Physiol Endocrinol Metab. 2014;307:E426–36. 10.1152/ajpendo.00264.2014. 25053401 10.1152/ajpendo.00264.2014 PMC4154070

